# Chloroquine Enhances Gefitinib Cytotoxicity in Gefitinib-Resistant Nonsmall Cell Lung Cancer Cells

**DOI:** 10.1371/journal.pone.0119135

**Published:** 2015-03-25

**Authors:** Mei-Chuan Tang, Mei-Yi Wu, Ming-Hung Hwang, Ya-Ting Chang, Hui-Ju Huang, Anya Maan-Yuh Lin, James Chih-Hsin Yang

**Affiliations:** 1 National Center of Excellence for Clinical Trial and Research, National Taiwan University, Taipei, Taiwan; 2 Institute of Pharmacology, National Yang-Ming University, Taipei, Taiwan; 3 Graduate Institute of Oncology, National Taiwan University, Taipei, Taiwan; 4 Department of Medical Research, Taipei-Veterans General Hospital, Taipei, Taiwan; 5 Graduate Institute of Oncology, National Taiwan University and Department of Oncology, National Taiwan University Hospital, Taipei, Taiwan; Seoul National University, REPUBLIC OF KOREA

## Abstract

Epidermal growth factor receptor tyrosine kinase inhibitors (EGFR-TKIs), including gefitinib, are effective for non-small cell lung cancer (NSCLC) patients with *EGFR* mutations. However, these patients eventually develop resistance to EGFR-TKI. The goal of the present study was to investigate the involvement of autophagy in gefitinib resistance. We developed gefitinib-resistant cells (PC-9/gef) from PC-9 cells (containing exon 19 deletion *EGFR*) after long-term exposure in gefitinib. PC-9/gef cells (B4 and E3) were 200-fold more resistant to gefitinib than PC-9/wt cells. Compared with PC-9/wt cells, both PC-9/gefB4 and PC-9/gefE3 cells demonstrated higher basal LC3-II levels which were inhibited by 3-methyladenine (3-MA, an autophagy inhibitor) and potentiated by chloroquine (CQ, an inhibitor of autophagolysosomes formation), indicating elevated autophagy in PC-9/gef cells. 3-MA and CQ concentration-dependently inhibited cell survival of both PC-9wt and PC-9/gef cells, suggesting that autophagy may be pro-survival. Furthermore, gefitinib increased LC3-II levels and autolysosome formation in both PC-9/wt cells and PC-9/gef cells. In PC-9/wt cells, CQ potentiated the cytotoxicity by low gefitinib (3nM). Moreover, CQ overcame the acquired gefitinib resistance in PC-9/gef cells by enhancing gefitinib-induced cytotoxicity, activation of caspase 3 and poly (ADP-ribose) polymerase cleavage. Using an *in vivo* model xenografting with PC-9/wt and PC-9/gefB4 cells, oral administration of gefitinib (50 mg/kg) completely inhibited the tumor growth of PC-9/wt but not PC-9/gefB4cells. Combination of CQ (75 mg/kg, i.p.) and gefitinib was more effective than gefitinib alone in reducing the tumor growth of PC-9/gefB4. Our data suggest that inhibition of autophagy may be a therapeutic strategy to overcome acquired resistance of gefitinib in *EGFR* mutation NSCLC patients.

## Introduction

Autophagy, known as a self-eating mechanism, is characterized by de novo synthesizing double-membrane autophagosomes which sequester cellular components such as excessive or unnecessary protein and organelles [1–3]. Fusion of autophagosomes with lysosomes reportedly degrades the cytosolic contents into essential components for recycle. Physiologically, a basal level of autophagy is vital for the cellular homeostasis. Furthermore, autophagy is reportedly induced to cope with stresses such as hypoxia as well as nutrient deprivation and considered as a survival strategy [1–3]. In contrast, a pro-death role of autophagy is proposed as a type II programmed cell death through over-activation of self-eating [4]. Indeed, autophagy inducers were found to reduce tumor volume [5–7]. However, inhibition of autophagy reportedly induced cancer cell death [8–10], suggesting that autophagy plays a cytoprotective role for cancer cells. In support of this notion, autophagy inhibition by 3-methyladenine (3-MA), chloroquine (CQ, a lysosomotropic agent to inhibit autophagolysosome formation) and autophagy (ATG)-related gene 5 silencing was found to augment the cytotoxic effects by chemotherapies and target therapy [11–16]. Accordingly, autophagy becomes a potential target for cancer treatments.

Drug resistance has been a focus of interest in the study of cancer therapy. Several lines of evidence have suggested the involvement of autophagy in drug resistance, both innate drug resistance and acquired drug resistance. For example, CQ has been shown to overcome primary resistance of epidermal growth factor receptor (EGFR) tyrosine kinase inhibitors (TKIs) in A549 lung cancer cells [16] and trastuzumab in HER-2 positive breast cancer [17]. Several *in vitro* studies have demonstrated that CQ and bafilomycin A1 restore the sensitivity to crizotinib and trastuzumab in acquired resistant cells, respectively [18–19]. Furthermore, 3-MA was found to enhance the cytotoxic effect of cisplatin in cisplatin-resistant cells [20], indicating that inhibition of autophagy appears to be a therapeutic target for acquired drug resistance.

Non-small cell lung cancer (NSCLC) is the most common cancer in the world. Currently, epidermal growth factor receptor (EGFR) tyrosine kinase inhibitors (TKIs), including gefitinib, erlotinib and afatinib, are highly effective in treating lung cancer patients with specific *EGFR* mutations in their tumor samples, such as exon 19 deletion or exon 21 L858R mutation [21–23]. Despite the success of using EGFR-TKIs in the treatment for East Asian NSCLC patients, all responding patients eventually developed acquired resistance to EGFR-TKIs [24–27]. In the present study, the involvement of autophagy in the acquired gefitinib resistance in *EGFR* mutation NSCLC cells was investigated using PC-9/wt cells carrying *EGFR* exon 19 deletion and the acquired gefitinib-resistant PC-9/gef cells (PC-9/gefB4 and PC-9/gefE3).

## Materials and Methods

### Reagents and antibodies

The chemicals used were gefitinib (a kind gift from Astrazeneca, Alderley Park, UK), chloroquine diphosphate (CQ; Sigma, St. Louis, MO, U.S.A.), 3-methyladenine (3-MA; Sigma), and Cremophor EL (Sigma). The primary antibodies included microtubule-associated protein 1 light chain 3 (LC3; Cell Signaling Technology, Beverly, MA, U.S.A., #2775), caspase 3 (Cell Signaling Technology, #9668), and PARP (Cell Signaling Technology, #9542), α-tubulin (Cell Signaling Technology, #2144) and β-actin antibody (Millipore, Bedford, MA, U.S.A.). The secondary antibodies were horseradish peroxidase-conjugated secondary IgG (Chemicon, Temecula, CA, U.S.A.).

### Development of gefitinib-resistant PC-9 cells

PC9/gefB4 and PC9/gefE3 cells were developed in our laboratory and published previously [26]. PC-9/wt cells, a human lung adenocarcinoma cell line harboring a deletion in exon 19 of *EGFR* [28], were cultured in a humidified atmosphere of 5% CO_2_ at 37°C in RPMI (Roswell Park Memorial Institute) media containing 10% fetal bovine serum, 4.5 g/L glucose, and 1% (v/v) penicillin/streptomycin. PC-9/wt cells were grown in culture media containing escalating concentrations of gefitinib. After 6 months of passages, cells that could grow in micromolar concentrations of gefitinib were kept in drug-free media for 2 weeks and were cloned. Two clones (PC-9/gefB4, and PC-9/gefE3) were obtained for future studies.

### Growth inhibition assay

The stock solutions of gefitinib and 3-MA were prepared in dimethyl sulfoxide while CQ was in ddsH_2_O. Fifteen hundred cells were placed in 96-well flat-bottomed plates and cultured for 24 h. To establish IC_50_ of gefitinib, various concentrations of gefitinib were included in the culture medium for 96 h. Using sulforhodamine B assay [29], cell viability was determined by dividing the absorbance values of treated cells to that of untreated cells. IC_50_ calculated from the concentration-response curve was defined as the concentration of gefitinib which 50% growth inhibition was obtained. For the effects of 3-MA and CQ, the growth inhibition was measured after 96-h incubation of 3-MA (0.1, 0.3 or 1 mM) or CQ (5, 10 or 15 μM). For the effect of CQ on gefitinib-induced cell death, the growth inhibition was determined after 96-h incubation of gefitinib plus CQ (5 or 10 μM).

### Western blot assay of proteins

To evaluate the involvement of autophagy of PC-9/wt and gefitinib-resistant cells, cells were treated with gefitinib plus 3-MA or CQ for 24 h. Treated cells were harvested, washed with phosphate buffered saline (PBS), and lysed in radioimmunoprecipitation assay (RIPA) lysis buffer containing 20 mM Tris HCl, 150 mM NaCl, 1% (v/v) NP-40, 1% (w/v) sodium deoxycholate, 1 mM Ethylenediaminetetraacetates (EDTA), 0.1% (w/v) sodium dodecyl sulfate polyacrylamide (SDS) and 0.01% (w/v) sodium azide (pH 7.5) for 20 min on ice. Lysates were then centrifuged at 12,000 rpm for 10 min, and the protein concentrations of supernatant were determined by BCA Protein Assay Kit. Protein samples (30 μg) were run on 12–13.5% SDS-polyacrylamide gel electrophoresis and then transferred onto a polyvinylidene difluoride (Bio-Rad, U.S.A.) at 90 V for 120 min. Blots were probed with primary antibodies overnight at 4°C. After primary antibody incubation, the membrane was washed and incubated with a secondary antibody (1:3000) for 1 h at room temperature. The immunoreaction was visualized using Amersham Enhanced Chemiluminescence (Amersham Pharmacia Biotech, Piscataway, NJ, U.S.A.). After this detection, the bound primary and secondary antibodies were stripped by incubating the membrane in stripping buffer (100 mM 2-mercaptoethanol, 2% SDS) at 50°C for 45 min. The membrane was reprobed with a primary antibody against β-actin (1:5000)/α-tubulin (1:5000).

### Fluorescent and Immunofluorescent staining assay

Autolysosomes staining: Cells were treated with gefitinib for 24 h and then medium was replaced by fresh medium containing 50 nM Lysotracker Red (LysoTR, LysoTracker Red DND-99, Invitrogen, Carlsbad, CA, U.S.A.) and incubated at 37°C for 30 min. Afterwards, cells were rinsed in PBS and fixed with 3.5% paraformaldehyde (in PBS) for 10 min, permeabilized with 0.5% Triton X-100 in TBS for 30 min, and treated with 2% BSA in TBS for 1 h, at room temperature. Samples then were incubated with LC3 antibody (1:200) overnight at 4°C, rinsed three times with 0.01% Triton X-100 in TBS, and incubated for 30 min with a secondary antibody (FITC-conjugated rabbit IgG at 1:250) at 37°C. Afterwards, cells were further stained with DAPI and observed under a confocal microscopy (Olympus FV1000, Olympus America Inc., Center Valley, PA, U.S.A.).

### Xenograft mouse model

Sixty- three 6-week-old male Balb/c nude mice, weighing 25–30g, were used. The animal protocol was approved by the Institutional Animal Care and Use Committee of Taipei Veterans General Hospital, Taipei, Taiwan. (Permit Number: 2011-037).Tumors were induced by injecting PC-9/wt and PC-9/gefB4 cells (10^7^ cells in 100 μl PBS) subcutaneously into the back of mice. To obtain the tumor growth curve, daily measurement of tumor was performed. Perpendicular diameter with a digital caliper and volumes were calculated by (length x width^2^)/2. When tumors grew to 200 mm^3^, mice were randomized to 4 groups orally treated with vehicle (10% Cremophor EL/10% ethanol/4% dextrose in ddH_2_O), gefitinib alone (50 mg/kg, by a gavage), CQ alone (75 mg/kg, i.p.) and gefitinib plus CQ. Gefitinib was prepared in 10% Cremophor EL/10% ethanol/4% dextrose and CQ was dissolved in PBS.

### Statistics

All data are expressed as the mean ± S.E.M. Statistical comparisons of cell viability were made using Independent-Samples T Test of SPSS. P value less than 0.05 was considered as statistically significant.

## Results

### Elevated basal autophagy in PC-9/gef cells

**Fig 1:**
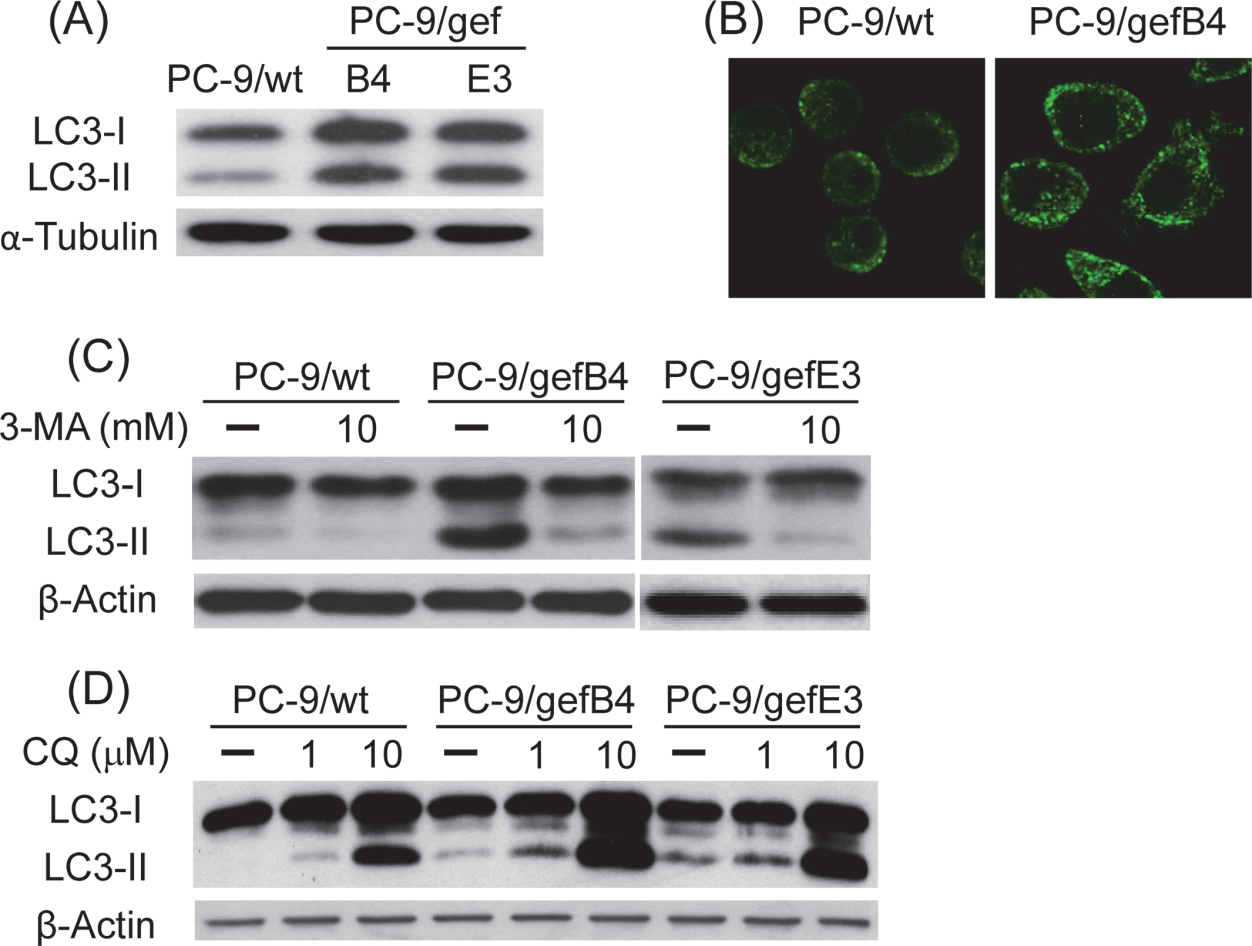
Basal autophagy levels in PC-9/wt and PC-9/gef cells. (A) Representative Western blot data showed the basal LC3-II levels in PC-9/wt and PC-9/gef cells (B4 and E3). (B) Immunofluorescent staining studies were performed using LC3 antibody to show LC3 puncta in the PC-9/wt and PC-9/gefB4 cells. (C and D): PC-9/wt, PC-9/gefB4 and PC-9/ gefE3 cells were treated with 3-methyladenine (3-MA, 10 mM) and chloroquine (CQ, 1 and 10 μM) for 24 h. Total protein of treated cells was harvested; LC3-I and II levels were analyzed with Western blot assay. Each lane contained 30 μg protein for all experiments. Results were repeated in independent experiments.

**Fig 2:**
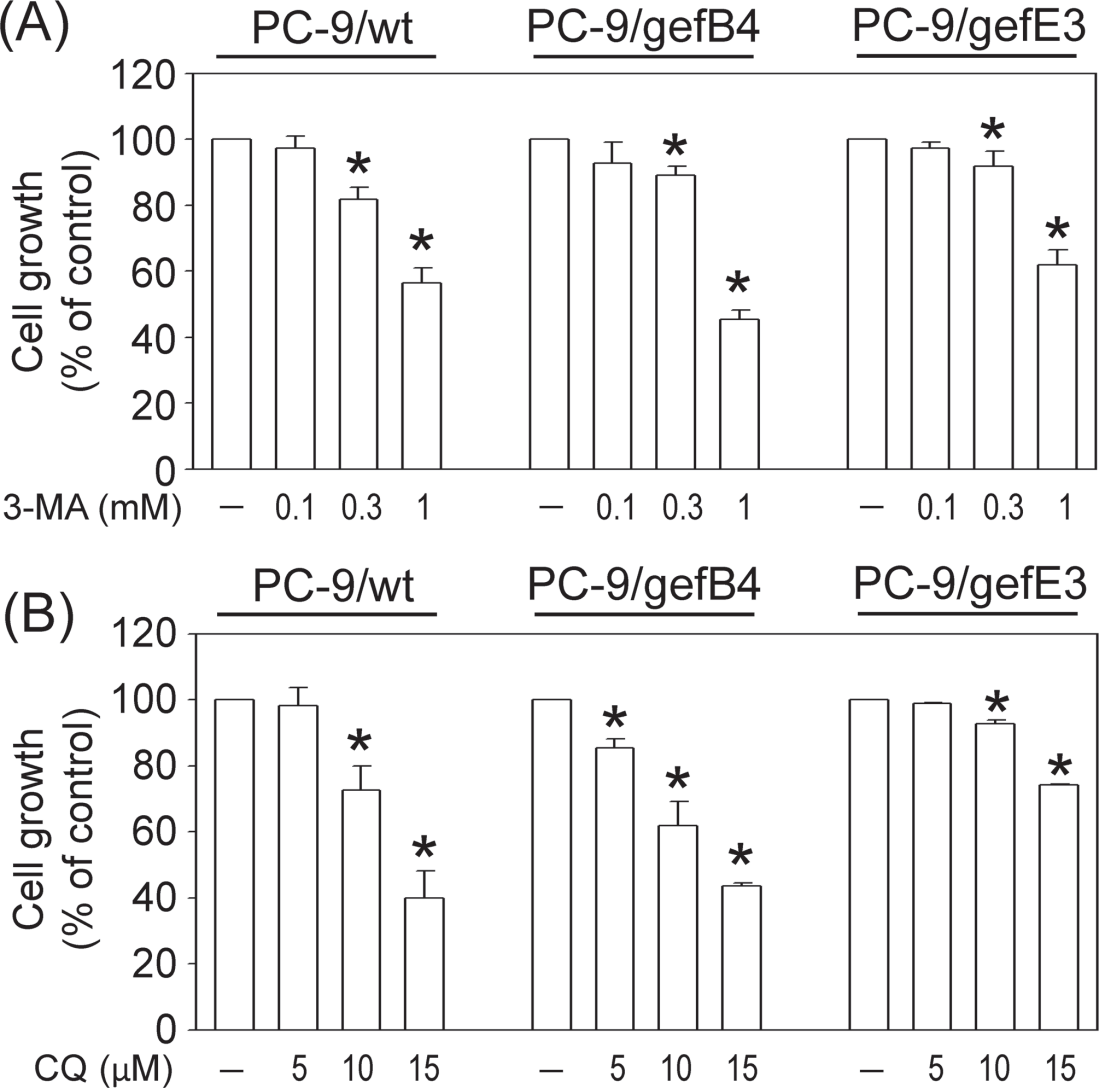
Effects of autophagy inhibitors on proliferation of PC-9/wt and PC-9/gef cells. PC-9/wt, PC-9/gefB4 and PC-9/gefE3 cells were treated with (A) 3-MA (0.1–1 mM) and (B) CQ (5–15 μM) for 96 h. Cell viability was determined using SRB assay. Values are the mean ± S.E.M. (n = 3). *P < 0.05 statistically significant in the 3-MA or CQ-treated groups compared with the controls.

To study autophagy and drug resistance, the level of LC3-II, a hallmark protein of autophagy, was measured in PC-9/wt, PC-9/gefB4 and PC-9/gefE3 cells. Western blot assay showed higher basal levels of LC3-II in PC-9/gef cells (B4 and E3) while compared with PC-9/wt cells (Fig. 1A). Consistent with the Western blot assay, the immunostaining study demonstrated more LC3 immunofluorescent puncta in PC-9/gefB4 cells compared with PC-9/wt cells (Fig. 1B). Furthermore, 3-MA and CQ were employed to characterize the autophagy. We found that 3-MA decreased (Fig. 1C) while CQ increased the basal LC3-II levels in PC-9/wt, PC-9/gefB4 cells and PC-9/gefE3 after 24-h drug treatments (Fig. 1D). These data indicate that PC-9/gef (B4 and E3) cells have a higher basal level of autophagy than PC-9/wt cells. Furthermore, the cell survival assay was employed to delineate the role of autophagy using 3-MA and CQ. SRB assay showed that 3-MA and CQ concentration-dependently reduced cell survival in PC-9/wt, PC-9/gefB4 and PC-9/gefE3 cells (Fig. 2), suggesting that autophagy plays a pro-survival role in cell proliferation.

### Gefitinib-induced autophagy *in vitro*

**Fig 3:**
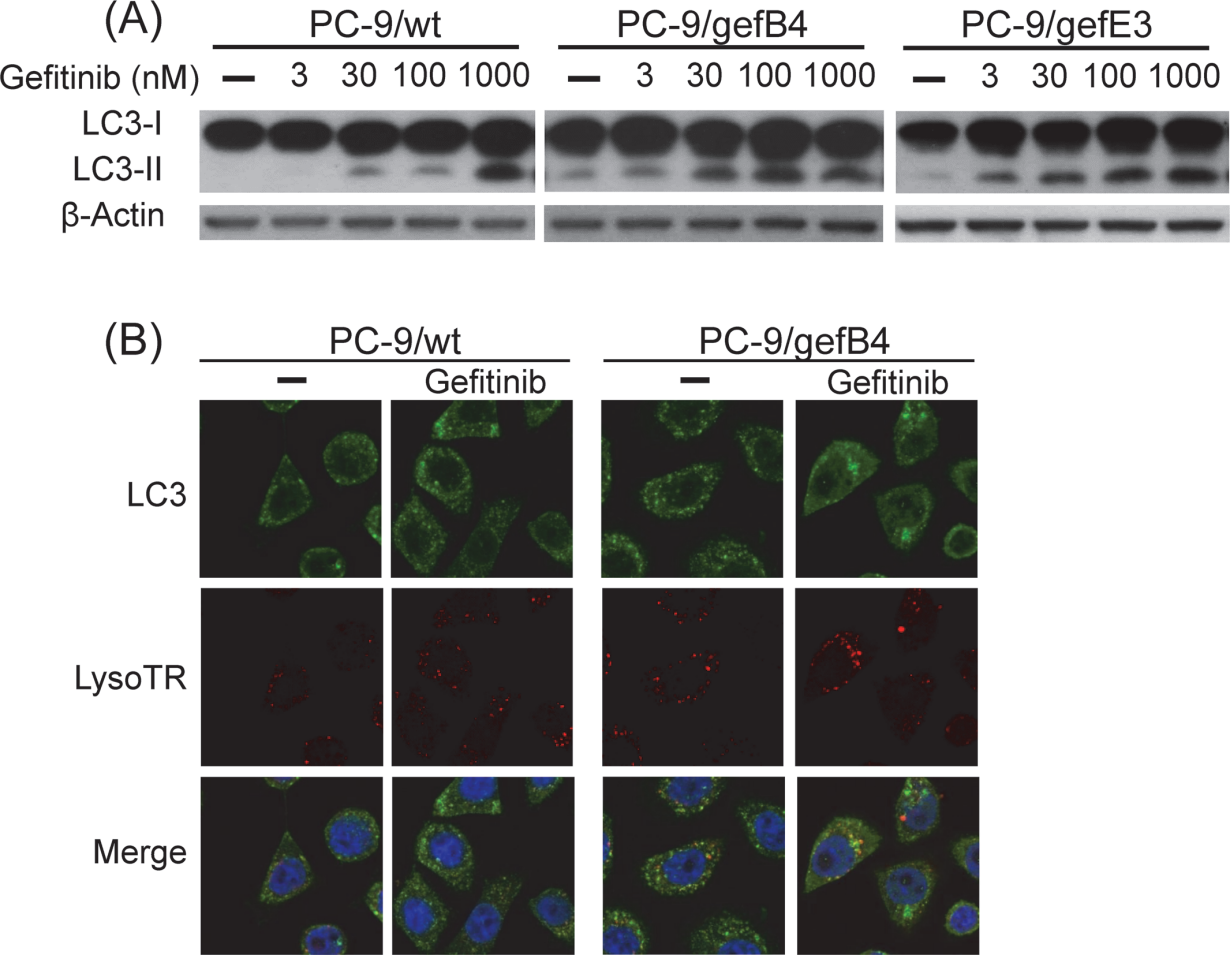
Effects of gefitinib on LC3-II level in PC-9/wt and PC-9/gef cells. (A) PC-9/wt, PC-9/gefB4 and PC-9/gefE3 cells were treated with gefitinib (nM) at various concentrations for 24 h. Total protein of treated cells was harvested; LC3-I and II levels were analyzed with Western blot assay. Each lane contained 30 μg protein for all experiments. Results were repeated in independent experiments. (B) PC-9/wt and PC-9/gefB4 cells were treated with gefitinib (1 μM) for 24 h. Immunofluorescent staining and fluorescent staining studies were performed using LC3 antibody and LysoTracker red (LysoTR) respectively to show puncta in the cells. Green, FITC-labeled LC3; red, LysoTR; blue, DAPI-labeled nucleus.

The involvement of autophagy in gefitinib-induced cytotoxicity was investigated. Western blot assay showed that gefitinib concentration-dependently increased LC3-II levels in PC-9/wt, PC-9/gefB4 and PC-9/gefE3 cells (Fig. 3A). Immunofluorescent staining studies demonstrated that 24-h incubation of gefitinib (1μM) elevated LC3 puncta in both PC-9/wt and PC-9/gefB4 cells (Fig. 3B). Furthermore, co-localization of LC3 immunofluorescence and LysoTR fluorescence was observed in gefitinib-treated PC-9/WT and PC-9/gefB4 cells (Fig. 3B), indicating that gefitinib is capable of inducing autophagy and autolysosome formation.

**Fig 4:**
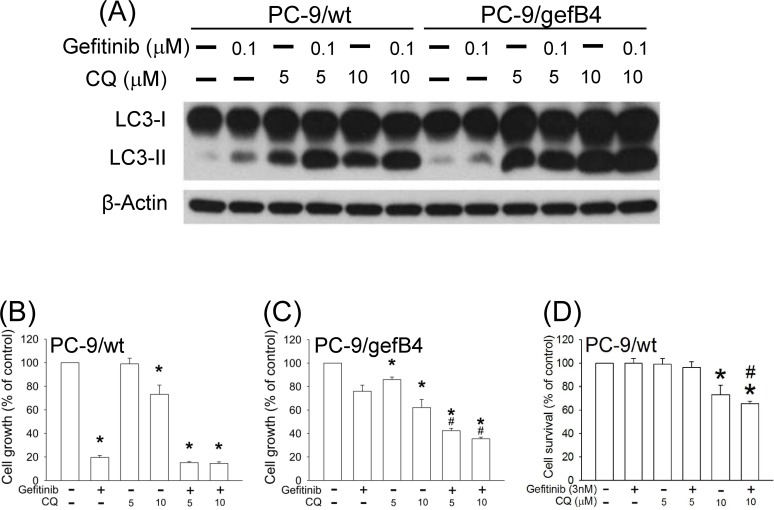
Autophagy activation and cytotoxicity by gefitinib and chloroquine in PC-9/wt and PC-9/gefB4 cells. (A) PC-9/wt and PC-9/gefB4 were treated with gefitinib (100 nM) and chloroquine (CQ, 5, 10 μM) for 24 h. Total protein of treated cells was harvested. LC3-II levels were analyzed with Western blot assay. Each lane contained 30 μg protein for all experiments. Results were repeated in independent experiments. (B) PC-9/wt and (C) PC-9/gefB4 cells were cultured with gefitinib (100 nM) and CQ (5, 10 μM) for 96 h. (D) PC-9/wt cells were cultured with gefitinib (3 nM) and CQ (5, 10 μM) for 96 h. Cell viability was determined using SRB assay. Values are the mean ± S.E.M. (n = 3). *P < 0.05 statistically significant in the gefitinib or CQ-treated groups compared with the controls; # P< 0.05 statistically significant in gefitinib plus CQ-treated groups compared with gefitinib only groups.

**Fig 5:**
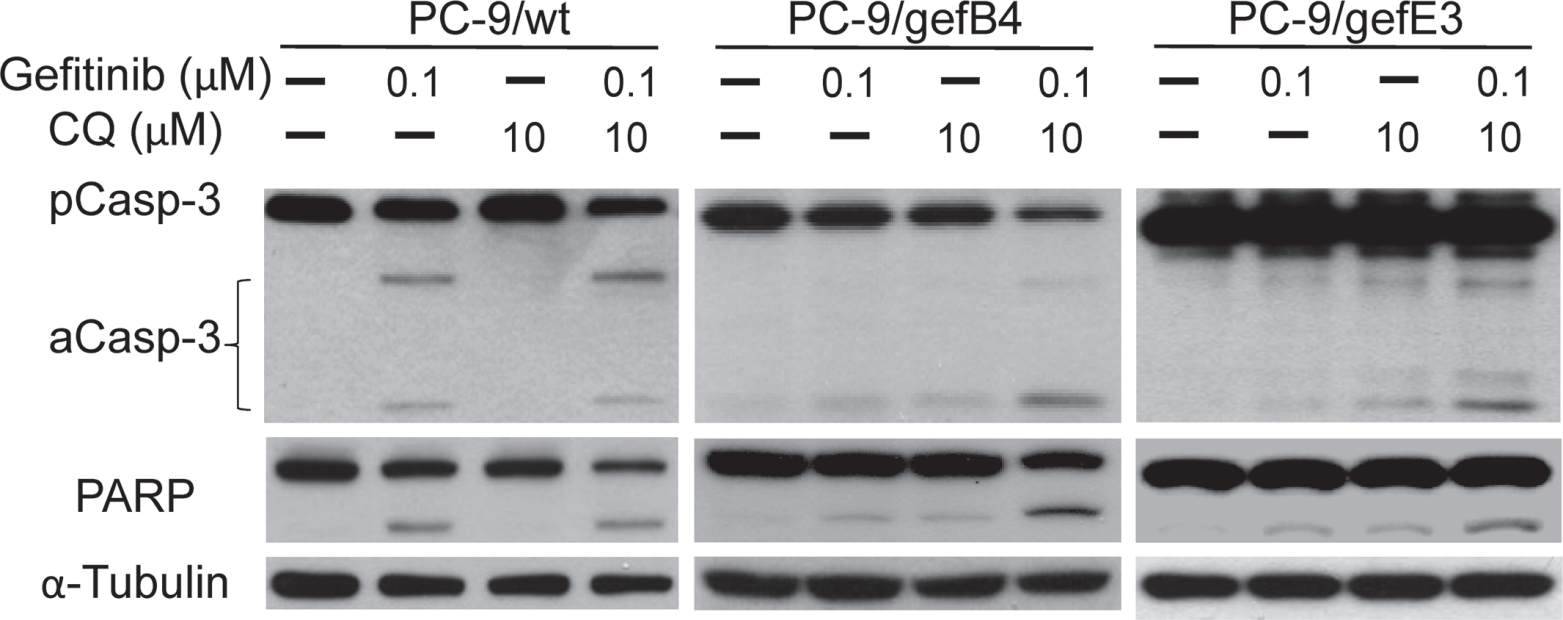
Activation of apoptotic pathway by gefitinib and chloroquine in PC-9/wt and PC-9/gef ells. PC-9/wt, PC-9/gefB4 and PC-9/gefE3 cells were treated with gefitinib (100 nM) and chloroquine (CQ, 10 μM) for 24 h. Total protein of treated cells was harvested. Procaspase 3, active caspase 3, PARP cleavage levels was analyzed with Western blot assay. Each lane contained 30 μg protein for all experiments. Results were repeated in independent experiments.

Due to the gefitinib-elevated autophagy, the effect of CQ on gefitinib-induced cytotoxicity was investigated. Twenty-four hours after drug treatments, CQ further increased gefitinib-induced LC3-II levels in PC-9/wt and PC-9/gefB4 cells (Fig. 4A). Cell survival studies demonstrated that gefitinib (100 nM) alone induced profound cell death of PC-9/wt cells; however, CQ (5 and 10 μM) was unable to further enhance gefitinib-induced cytotoxicity (Fig. 4B). As to PC-9/gefB4 cells, gefitinib (100 nM) alone induced a slight cell death; CQ significantly potentiated the gefitinib-induced cytotoxicity (Fig. 4C), i.e., CQ overcame gefitinib resistance in PC-9/gefB4 cells. To further test the potentiation of CQ in PC-9/wt cells, low dose of gefitinib (3 nM) was employed. While 96-h incubation of gefitinib (3 nM) induced an insignificant cytotoxicity in PC-9/wt cells, co-incubation with CQ (10 nM) and gefitinib profoundly reduced the cell survival (Fig. 4D). The CQ-induced potentiation of gefitinib-induced cytotoxicity was further investigated by measuring apoptosis-related proteins, including caspase-3 and poly (ADP-ribose) polymerase (PARP) levels. We found that gefitinib (0.1 μM) induced apoptosis by showing caspase 3 activation and PARP cleavage in PC-9/wt cells; CQ (10 μM) consistently did not augment gefitinib-induced apoptosis in PC-9/wt cells (Fig. 5). In contrast, when gefitinib or CQ alone was unable to induce apoptosis in PC-9/gefB4 and PC-9/gefE3 cells, CQ plus gefitinib significantly induced caspase 3 activation and PARP cleavage in both PC-9/gef cells (Fig. 5).

### Gefitinib plus CQ potentiated gefitinib-induced anti-tumor activity in PC-9/gefB4 xenografts

**Fig 6:**
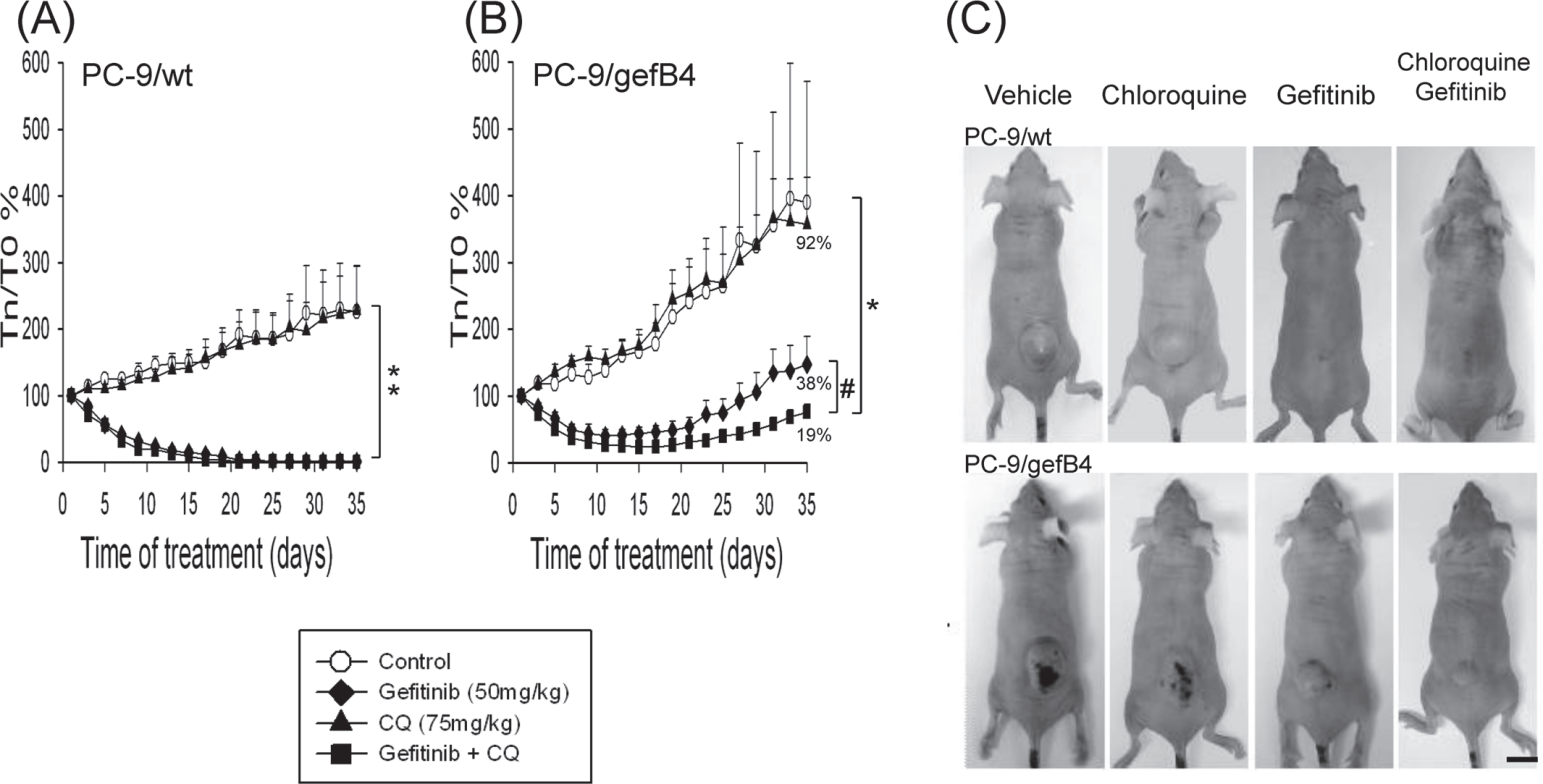
The anti-tumor effect of gefitinib and chloroquine in the mouse xenograft model. Balb/c nude mice bearing PC-9/wt (A) and PC-9/gefB4 (B) xenografts were treated with vehicles as control (○, n = 4 for PC-9/wt and PC-9/gefB4, respectively), gefitinib (50 mg/kg/day by a gavage,◆; n = 5 for PC-9/wt and PC-9/gefB4, respectively), chloroquine (CQ, 75 mg/kg, i.p. ▲; n = 4 for PC-9/wt and PC-9/gefB4, respectively), or a combination of both (■; n = 5 for PC-9/wt and PC-9/gefB4, respectively).Tumors were allowed to grow to 200 mm^3^ before drug treatments. Values are the mean ± S.E.M. (n = 4–5). ** p<0.001, statistically significant in gefitinib and gefitinib plus CQ groups compared with the vehicle group in PC-9/wt tumor xenografts. * p<0.05, statistically significant in gefitinib plus CQ group compared with the vehicle group; # p<0.05 statistically significant in gefitinib plus CQ group compared with gefitinib alone in PC-9/gefB4 tumor xenografts by Independent-Samples T Test. (C) Representative data show 4 mice with PC-9/wt xenografts and 4 mice with PC-9/gefB4 xenografts which were treated with vehicle, CQ only, gefitinib only and gefitinib plus CQ.

The CQ-induced potentiation of the anti-tumor activity of gefitinib was further investigated using Balb/c nude mice with PC-9/wt and PC-9/gefB4 human tumor xenografts (Fig. 6). CQ alone did not alter the tumor growth of PC-9/wt and PC-9/gefB4 human tumor xenografts (Fig. 6). Compared with the vehicle-treated tumor growth, gefitinib monotherapy significantly inhibited the tumor growth of PC-9/wt xenografts; co-administration of CQ was unable to augment the anti-tumor effect by gefitinib in PC-9/wt xenografts (Fig. 6A). The complete inhibition of tumor growth of PC-9/wt xenografts lasted until the end of experiment (Fig. 6A). In contrast, gefitinib insignificantly reduced tumor growth of PC-9/gefB4 xenografts compared with that of PC-9 xenografts (Fig. 6B; p = 0.07). PC-9/gefB4 tumors re-grew after 15 days of gefitinib administration (Fig. 6B). Surprisingly, CQ plus gefitinib significantly suppressed the tumor growth of PC-9/gefB4 xenografts compared with gefitinib only (Fig. 6B; p<0.05).

## Discussion

### Autophagy and drug resistance

To develop therapeutic strategies for the acquired resistance induced by gefitinib, we used PC-9/gefB4 and PC-9/gefE3 cells which possess IC_50_ of gefitinib approximately 200-fold more than that of PC-9/wt cells [26]. Western blot assay and immunofluorescent study demonstrated higher basal levels of autophagy in both PC-9/gefB4 and PC-9/gefE3 cells. Using 3-MA and CQ to impair formation and function of autophagy, the mechanism for the elevated basal autophagy in gefitinib-resistant cells is proposed. To cope with unfavorable stresses, i.e., constant exposure to gefitinib, cancer cells increased autophagy to maintain metabolic homeostasis and appropriate cell growth [4]. Cell viability assay revealed that autophagy was pro-survival because 3-MA and CQ decreased cell survival of PC-9/wt and PC-9/gef cells (B4 and E3). In addition to gefitinib resistance, previous studies have reported that trastuzumab-refractory breast cancer cells and cisplatin-resistant lung cancer cells have higher autophagy compared with the sensitive cancer cells [19–20]. Similarly, 3-MA and bafilomycin A1 (an inhibitor of autolysosome maturation) were found to reduce cell viability of these resistant cells [19–20]. In contrast to the 200-fold difference in IC_50_ of gefitinib [26], 3-MA and CQ induced cell death to a similar extent in PC-9 and PC-9/gef cells, indicating PC-9/gefB4 cells were not resistant to 3-MA and CQ-induced cytotoxicity.

### Role of autophagy in gefitinib-induced cytotoxicity

Different therapies including radiation therapy [20], chemotherapies [30] and target therapies [16, 31] have been reported to induce autophagy. Our study confirmed this notion that gefitinib increased autophagy in a concentration-dependent manner in PC-9/wt and PC-9/gef cells (B4 and E3). The role of autophagy in gefitinib-induced cytotoxicity was further delineated by combination of CQ and gefitinib. Consistent to our previous study [26], gefitinib (100 nM) alone induced marked cytotoxicity in PC-9/wt cells. The cytotoxic mechanism of gefitinib is known to compete the ATP binding site in PC-9/wt cells carrying the *EGFR* exon 19 deletion [32] and thus induce cytotoxicity through apoptosis [26, 33–35]. The potentiation by CQ plus gefitinib in PC-9/wt cells was observed only when gefitinib was reduced to 3 nM, indicating that CQ may be used to augment gefitinib-induced apoptosis in PC-9/wt cells. One clinical trial of gefitinib and hydroxychloroquine is undergoing on advanced NSCL patients patients [36], our data suggest that when co-administrating with CQ and its analogs, lower doses of gefitinib may be enough for patients having gefitinib-sensitive lung cancers with less side effects [37].

### Autophagy and acquired drug resistance

Compared with PC-9/wt cells, a 200-fold difference in IC_50_ of gefitinib was identified in PC-9/gefB4 cells [26]. Our previous study found that MEK inhibitors (AZD6244 and CI1040) profoundly reversed the acquired resistances to gefitinib in PC-9/gefB4 cells [26]. Consistently, gefitinib (100 nM) alone was unable to induce significant cytotoxicity in PC-9/gefB4 (Fig. 4C), PC-9/gefE3 and PC-9/gefE7 [26]. However, the present study showed that gefitinib plus CQ significantly induced caspase 3 activation and PARP cleavage in both PC-9/gefB4 and PC-9/gefE3 cells, indicating that CQ sensitized PC-9/gef cells (B4 and E3) to gefitinib. Compared with the 200-fold difference in IC_50_ of gefitinib, gefitinib did not show significant differences in LC3-II elevation and cell death in PC-9/wt cells, PC-9/gefB4 and PC-9/gefE3 cells, indicating autophagy may not be responsible in the acquired gefitinib resistance. Nevertheless, our *in vitro* data showed that CQ attenuated survival of PC-9/gefB4 cells, indicating that gefitinib and CQ may be effective to overcome gefitinib resistance. In addition, several *in vitro* studies reported that autophagy inhibition appears to enhance cytotoxicity in the crizotinib-resistant cells [18], trastuzumab-resistant cells [19] and cisplatin-resistant cells [20]. So far, limited *in vivo* studies have focused on the therapeutic effect of CQ on acquired drug resistance [18]. Our findings from *in vivo* xenograft model support the *in vitro* data in that gefitinib consistently inhibited PC-9/wt tumor growth and CQ did not enhance the anti-cancer effect of gefitinib. As to the tumor growth of PC-9/gefB4 xenografts, CQ plus gefitinib significantly delayed the tumor growth of PC-9/gefB4 xenografts compared with gefitinib monotherapy, indicating that CQ is capable of sensitizing the PC-9/gefB4 cells to gefitinib and then reduces tumor growth of PC-9/gefB4 human xenografts.

In conclusion, EGFR-TKIs, such as gefitinib, are known to treat lung cancers with significant efficacies. Our previous study employed combination of gefitinib and ERK inhibitors and successfully demonstrated significant therapeutic potentials for the acquired resistance to gefitinib [26]. In the present study, we showed that autophagy may play a cytoprotective role in the tumorigenesis and acquired resistance. Furthermore, CQ appears to be therapeutically useful for both gefitinib-sensitive and -resistant NSCLC, suggesting that CQ and its analogs may be a promising cancer therapy [38–39] for lung cancer patients with *EGFR* mutation who develop an acquired resistance after receiving gefitinib treatment.
